# Woven solutions for tissue engineering: Next-generation heart valves from fiber to function

**DOI:** 10.1016/j.ahjo.2025.100604

**Published:** 2025-09-10

**Authors:** Cornelia Sennewald, Jasmin Pilgrim, Dilbar Aibibu, Thomas Gereke, Philipp Schegner, Chokri Cherif

**Affiliations:** a Institute of Textile Machinery and High Performance Material Technology (ITM), Technische Universität Dresden, Hohe Straße 6, 01069, Dresden, Saxony, Germany

**Keywords:** Prosthesis design, Biomedical engineering, Biomedical technology, Computer-aided design, Textiles, Models, Cardiovascular

## Abstract

**Study objective:**

Cardiovascular diseases remain one of the leading causes of morbidity and mortality worldwide, yet the availability of durable, patient-specific heart valve replacements is still limited. The aim is to utilize a biomimetic, textile-based design to mimic natural tissues, thereby creating customizable solutions with improved mechanical properties and scalable production for cardiovascular applications.

**Design:**

By leveraging advanced 3D weaving techniques, the feasibility of manufacturing anatomically adaptable and mechanically robust textile valves is demonstrated. CAD-based design workflows and functional materials such as shape memory Nitinol wires are part of this technology. The integration of form-defining geometries, multilayer structures and functional surface treatments is enabled through tailored binding design and machine adaptations.

**Main outcome and results:**

A textile-based heart valve implant was developed using advanced 3D weaving, CAD modelling and patient-specific imaging. Integrated leaflets and an annular ring were formed directly during weaving using mold inserts and multilayer structures, eliminating post-processing. Polyester and Nitinol materials provided mechanical stability and shape-memory functionality. Simulation models and SPH analysis validated pressure behaviour and deformation under physiological conditions. Functional zones with tailored stiffness, sealing and mobility were realized through binding variation and Jacquard control. A reproducible digital workflow, from CT segmentation to weaving on modified looms, enabled scalable production of anatomically accurate, functionally optimized heart valve prostheses. Mechanical evaluations reveal favourable performance in comparison to conventional valve designs.

**Conclusion:**

These findings highlight the potential of fiber and textile technology as a scalable, customizable clinically relevant platform for heart valve tissue engineering and future biomedical applications.

## Highlights

• Multilayer weaving enables integrated zones with tailored mechanical properties
• Bicuspid and tricuspid walves can be woven in a single multilayer weaving process
• Hierarchical tissue architecture is mimicked via targeted textile structure design
• CT-based workflow allows fully patient-specific heart valve prothesis design
• Custom weaving and simulation enable precise control over valve leaflet dynamics

## 1 Introduction

The increasing life expectancy and the higher activity level of Western population are giving rise to continuous new medical challenges [1,2]. These include the rising incidences of cardiovascular diseases which led to a 23 % increase in the demand for implant solutions and surgical interventions between 2008 and 2018 in Germany [3]. In 2023, 37 % of all deaths in Germany were attributed to cardiovascular diseases, corresponding to 384,312 cases [4]. In the same year, the average prevalence of cardiovascular diseases was 9.99 % among men and 6.12 % among women, with a significantly higher incidence in older age groups [5]. Globally, between 2010 and 2019, the number of people affected by cardiovascular diseases increased by 26,6 %, reaching 523.2 million cases [6].

Over the course of an average human lifespan of 79.1 years (Europe) [2], the heart pumps approximately 2.5 billion times, circulating between 200 and 250 million liters of blood through the human body, thereby placing considerable strain on the heart valves. These valves ensure unidirectional blood flow; however; when they fail to close properly (insufficiency) or become narrowed and stiff (stenosis), blood flow is impaired. A consequence of these conditions, approximately 35,877 heart valve procedures were performed in Germany in 2018. In addition 24,233 heart valves were replaced [3]. Given the high mortality rate associated with these diseases, the availability of implants for heart valve replacement plays a significant societal and medical role.

Current aortic valve replacements (AVR) with biological valves key limitations, as do synthetic ones. The Ross procedure [7] involves the diseased aortic valve with autologous pulmonary valve while the latter is substituted with an allograft valve. This approach eliminates the need of long-term anticoagulation and is therefore attractive for young persons, especially women with fertility wish; however the risk of recurrent diseases or structural degeneration over time remains. Furthermore, the procedure is technically complex and entails the replacement of two valves despite only one being initially diseased. Other biological heart valves exhibit the same risk of degeneration over time and reoperations [8]. Synthetic implants on the other hand exhibit a lower risk of reoperation but can trigger inflammation, produce audible clicks [9] that can lead to psychological anxiety disorders [10] or degrade inconsistently with healing.

At this point, a clear research gap becomes evident regarding artificial heart valve prostheses that combine the advantages of mechanical heart valves such as reduced risk of reoperation, long durability and high biocompatibility with those of biological heart valves, including optimal adaptation to the surrounding tissue, individualized sizing and favourable hemodynamic performance.

To overcome this research gap, this study addresses alternative biomimetic materials that are already promising in the field of vascular replacement. Biomimetic designs are gaining traction, aiming to replicate natural tissue properties, such as graded stiffness, functionalized surfaces and resorption aligned with healing. For designing nature-inspired implants nature itself offers a model: Native tissue like tendons, ligaments, vessels and bones are made of hierarchically structures from molecular fibers to macrostructures, a principle mirrored in textiles, where fibers form yarns, yarns form fabrics and the weave defines mechanics.

Textile technology thus provides a strong foundation for complex, functional, patient-specific implants. Woven structures enable precise mechanical tuning within a single component, offering tailored elongation zones, directional strength, or integrated branching. With advanced CAD tools, medical imaging and digital weaving technologies, anatomical data can now be translated into customized textile implants with high precision and repeatability.

To streamline development of alternative biomimetic AVR, a modular solution matrix linking structure, function and indication is proposed. Combined with a reproducible process chain, this can shorten development times, reduce costs and accelerate innovation by recombining proven structures into new designs. This study aims to address the research gap by introducing a concept for the development of a woven, form-adaptive heart valve prototype, integrating CAD design, weave development, machine adaptation and functionalization – highlighting both the technical and medical potential of textile-based implants like hierarchical structure design, functionalizable surfaces, long durability without closing sounds and focus on hemodynamic performance.

## 2 Methods

### 2.1 Material selection and functionalization

For the AVR prototypes, polyethylene terephthalate (PET) was used as the primary filament material, processed in monofilament form, ensuring consistent mechanical properties across warp and weft directions and enabling precise fabric control. This Material can be replaced in subsequent studies by medically preferred filament-based materials that meet the requirements of textile processing using a weaving machine. When selecting the textile material, factors such as surface properties, the potential for endothelization and biocompatibility may serve as criteria.

To enhance biocompatibility and promote endothelialization, collagen coatings were applied to selected implant regions. The combination of PET and biologically active surfaces aimed to ensure both mechanical and physiological performance.

To ensure the shape stability of the artificial woven heart valve a Nitinol wire is used in the circumferential direction. The design thus follows the state of the art for stent grafts. Nitinol, a nickel‑titanium alloy, is characterized by an excellent shape memory effect [11], which allows the prosthesis to expand in a defined manner after minimally invasive implantation [12]. In addition, Nitinol wires are biocompatible [13], superelastic [14] and exhibit long-term mechanical stability [15], which is necessary for use in dynamically loaded vessels.

Zonal functionality, achieved through variation of yarn type, density and binding, was essential for replicating the graded stiffness and anisotropic elasticity of native tissues.

### 2.2 Limitations of current implant solutions and the potential of hierarchical textile design

Despite advances in tendon and ligament prostheses, current solutions face major challenges. Allografts are limited in availability and may trigger immune responses and mismatched stiffness gradients, increasing the risk of re-rupture [[16], [17], [18], [19], [20], [21], [22], [23]]. The Ross procedure uses the autologous pulmonary valve as a replacement for the aortic valve while the latter is replaced by a homograft. Pulmonary and aortic valve are very similar in structure, allowing the pulmonary valve to take over the function. This approach combines the advantages of mechanical biological prosthesis: It eliminates the need for anticoagulation medication and offers good durability, making it particularly beneficial for young persons, women who wish to have children [24]. The method is also applicable in children, as there are currently no implants small enough that can also grow over time [25,26].

Synthetic implants have the advantage of an almost unlimited durability but can cause inflammation and delayed healing due to degradation by-products and mismatched resorption rates. They also fail to reproduce the anisotropic mechanics of native tissue, leading to issues like creep and fatigue [[27], [28], [29], [30], [31], [32], [33], [34]]. In young and active individuals their main disadvantage is the requirement for lifelong anticoagulation [25].

A further variant consists of semi-synthetic heart valve prosthesis for minimally invasive use. These are balloon-expandable or self-expandable structures equipped with animal pericardium as the valve substitute [35,36]. Glutaraldehyde fixation of the tissue is commonly used, which promotes structural calcification. Studies have demonstrated a direct correlation between immune reactions and calcification [37]. Decellularization can reduce calcification, although an immune reaction cannot be completely ruled out in this case either [38].

These challenges highlight the limitations of currently available autografts, allografts and xenografts, underscoring the need for implants that combine mechanical and biological compatibility with patient-specific adaptability. Biomimetic approaches incorporating graded stiffness, tailored resorption and functionalized surfaces offer promising solutions. The central challenge lies in designing implants that fulfill both structural and biological roles, such as supporting tissue integrity and individual size. A promising strategy is the custom design of implants to match anatomical and mechanical demands.

Biomimetic approaches, such as graded stiffness, tailored resorption and functionalized surfaces, provide pathways to achieve these properties. Custom implant design that matches anatomical and mechanical needs is key to fulfilling structural and biological roles.

Human tissues from muscle to bone share a hierarchical organization starting with collagen fibrils [39], mirrored in textiles: fibers form yarns, yarns are interlaced into fabrics and binding determines macroscopic properties like stiffness and elasticity. This multiscale design logic enables modular implant development: material, fiber, yarn and binding form a toolbox for tailoring biological performance. Weaving technologies, when adapted at the material and machine level, can reproduce such complexity. Deconstructing existing textile solutions into key modules, structural characteristics, functional outcomes and application-specific requirements allows systematic recombination into novel, patient-specific implants. The result: textile-based implants that closely mimic native tissue behaviour across scales.

### 2.3 Structured design and process chain for fiber-based implants

Biomimetic implant development follows a structured, multi-step process, beginning with a detailed analysis of the biological tissue to be replaced, focusing on geometry and mechanical performance. From this analysis, specific implant requirements are defined, appropriate materials and textile structures are selected and digital models are developed and optimized. The entire process, including fabrication and validation, is conducted at the Institute of Textile Machinery and High Performance Material Technology (ITM).Patient-specific imaging data, such as CT scans, are used to define individual geometries and tolerances. Mechanical parameters such as tensile strength, stiffness, porosity, pressure resistance and motion, are systematically evaluated. This evaluation enables the identification of functional zones within the implant that require tailored properties, such as stiffness gradients, elasticity, sealing, or resorption.

To meet this requirements, standard weave structures are selected: multilayer weaves provide thickness, continuous weft threads enhance edge stability, locally varied warp density allow variable porosity and directional reinforcements are achieved through warp-weft interlocking or diagonal thread insertion. These elements collectively enable precise functional and mechanical customization. A digital solution matrix connects the defined functional requirements to structural options. These target specifications guide CAD modelling, yarn selection and segmentation into performance zones, which are then translated into binding patterns and control files for the customized weaving machine. After fabrication, the implant is evaluated against its geometric and mechanical specifications. Material or structural adjustments are made as necessary. The outcome is a reproducible, patient-specific textile implant optimized for biological integration and long-term function. This modular, digitally driven workflow not only facilitates the rapid development of customized woven heart valves but also establishes a foundation for future textile-based biomedical devices.

### 2.4 Characterization of native tissue properties and functional demands

Native tissue exhibits a hierarchical structure comparable to textile constructs [39]. This multiscale organization, from cells and the extracellular matrix to fiber bundles, enables precisely tuned mechanical properties and specialized functions. Heart valves exemplify such complex, multi-layered systems, making them a particularly relevant reference for the design of advanced textile-based implants.

To meet their functional demands, heart valves combine hydromechanical performance with intricate geometry. They comprise three main layers: the collagen-rich fibrosa for tensile strength, the spongiosa for damping and the ventricularis for elasticity. An endothelial lining promotes smooth blood flow and regulates molecular exchange. Being avascular, valves rely on diffusion from the bloodstream and are anchored to the heart wall by a fibrous annular ring that provides structural stability [40].

Operating via pressure gradients between heart chambers and the aorta, valves cycle at approximately 70 beats per minute [41]. They must endure peak pressures of up to 16 kPa, ensure complete closure to prevent regurgitation and accommodate pulsatile flow with wave-like leaflet motion. Functionally, they are categorized as semilunar (aortic, pulmonary) or atrioventricular (mitral, tricuspid) valves. The latter employ chordae tendineae and papillary muscles to prevent prolapse during ventricular contraction.

### 2.5 From anatomy to fiber-based implant: Digital workflow and implementation

A structured digital-to-textile workflow was established to develop anatomically and functionally optimized implants. Anonymized CT datasets were segmented using Amira® and 3D Slicer to extract anatomical structures such as the valve annulus, which were converted into polygonal surface meshes (STL) and refined in SolidWorks to define patient-specific geometries. These models were subdivide into functional zones such as leaflets, commissures and annulus.

Zones were unfolded into 2D layouts and translated into textile structures in EAT DesignScope, enabling binding design, yarn path planning and integration of functional elements such as Nitinol wires. Geometric compensation for shrinkage and deformation ensured anatomical fidelity.

Bindings patterns were tailored to local demands: high-strength twill or network weaves for anchoring zones and flexible satin or plain weaves for leaflet mobility. TexMind simulations and pre-tensioning models predicted yarn elongation, leaflet dynamics and load-deformation behaviour, guiding design choices.

Fabrication was performed on a modified MAGEBA band loom with independent warp let-offs, an electronic Jacquard (UNIVAL 100), a decoupled beating system with adjustable stroke and force and a custom leaflet-forming insert integrated into the reed.

This configuration enabled the direct weaving of anatomically curved leaflets in a continuous textile, eliminating post-processing and enhancing reproducibility. The integration of digital modelling, simulation and adaptive weaving allowed the production of complex, multilayer implants with zone-specific mechanics tailored to patient anatomy and function.

## 3 Results

### 3.1 Woven Fiber-based heart valve implant

A textile-based heart valve implant with anatomically integrated leaflets and an annular ring was developed using advanced 3D weaving. The Leaflets were directly formed during weaving via a mold insert, eliminating the need for post-processing and enabling the curved geometries essential for valve function.

Textile valves show strong potential for applications requiring unidirectional flow, such as in the heart. However, previous designs have lacked scalability and dynamic optimization. Inspired by native valve characteristics, such as flexibility, passive pressure-driven motion and durability, key design targets were defined. Polyester fibers were selected for their mechanical reliability, while shape-memory Nitinol wires were embedded to support folding and controlled deployment. Multilayer and form-weaving techniques allowed seamless integration of functional zones, including the leaflets and annulus. The use of a Jacquard module allowed precise variation in binding patterns.

A parameterized CAD model, based on patient-specific imaging data, ensured reproducible and anatomically tailored designs. Material selection was guided by mechanical, fluidic and surgical requirements including durability, sealing, fatigue resistance and suitability for minimally invasive procedures. Clinical input confirmed polyester fibers as appropriate, with Nitinol wires enabling shape recovery. All materials were characterized and matched to textile and structural criteria.

**Fig. 1:**
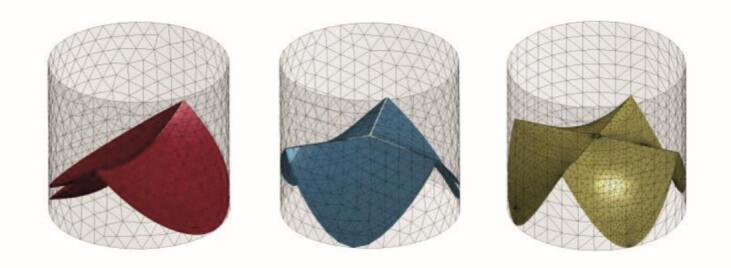
Parametric valve geometry with two (left), three (centre) and four (right) leaflets based on CAD modelling.

Shuttle weaving with continuous weft yarns was employed to create pressure-resistant tubular structures with reinforced edges [42]. Internal valve elements were formed using multilayer weaves, while pleated zones enabled controlled length variation for effective closure. The Jacquard mechanism provided patterning flexibility across the implant. Yarn fineness, weave density and binding structures were tailored to optimize mobility, sealing and surface properties. Based on implant parameters such as diameter, wall thickness and leaflet shape, a 3D CAD model was generated from patient-specific CT data and processed using custom software. This process produced a parameterized graphical textile layout (Fig. 1) to guide implant design.

**Fig. 2:**
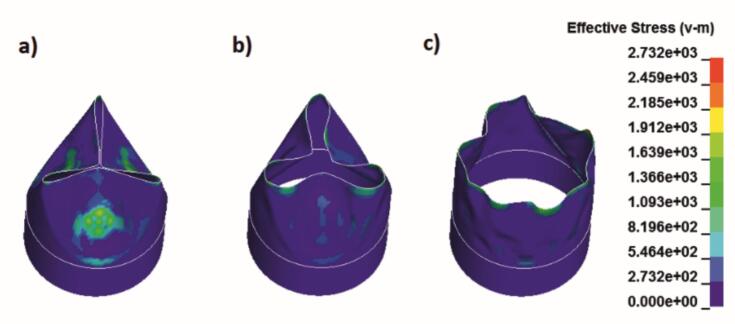
Explicit Deformation Simulation of the Valve Structures (a: closed state, b: during opening, c: open state).

Based on the geometric design, the heart valve was modelled using shell elements in a three-layer laminate configuration with integrated Nitinol wires to provide structural support and controlled deformation. To evaluate its mechanical performance under physiological conditions, in vitro tests were conducted in a hydrodynamic setup. High-speed imaging was used in combinations hydrodynamic data to capture aortic and ventricular pressures and flow rates over a 0.854-s cardiac cycle at 70 bpm. The recorded data enabled a detailed analysis of the valve's deformation behaviour throughout the cardiac cycle (Fig. 2).

The shell-based simulation model captures the anisotropic deformation behaviour of the textile structure. Nitinol reinforcement within the cylindrical tube provides structural support, eliminating the need for additional stiffening in the valve region. The simulation allows the identification of mechanically critical zones and, by incorporating textile parameters, enables targeted optimization of the weave design. The resulting simulation data can be directly compared with flow analysis measurements to access functional performance.

**Fig. 3:**
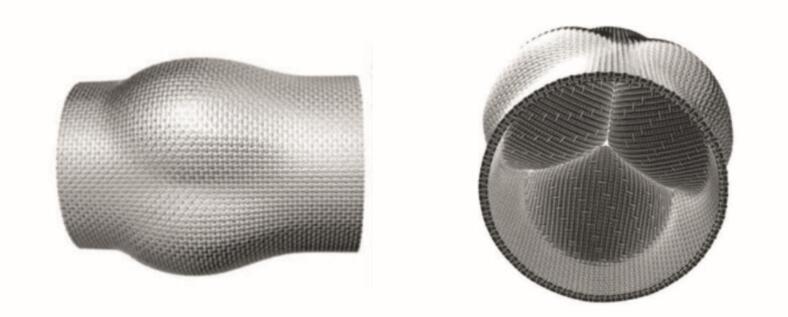
Seamless textile tube structure with integrated valve function, inspired by a heart valve prosthesis (left: top view; right: cross-section with valve and leaflet sealing surface).

To translate the simulation model into a textile implant, it is first unfolded into a two-dimensional layout. Weaving then enables 2.5D structures, which can be subsequently shaped. Multiple fabric layers can be produced simultaneously and selectively interconnected, a feature that is essential for integrating internal leaflets without the need for additional joining steps. Fig. 3 illustrates a woven valve model with clearly defined internal leaflet structures, emphasizing the importance of a multilayer design for functional fidelity.

To convert the simulation model into a textile structure, its orientation within the weaving process must first be defined. In shuttle weaving, two configurations are possible: (1) aligning the valve structure parallel to the weaving direction, or (2) orthogonal to it. For designs with fixed, closed tube ends and optimized leaflet length, only the orthogonal arrangement is suitable.

For the integration of Nitinol wires, shuttle weaving is combined with pleat weaving techniques. The pleat is formed by controlled floating of the outer layer across the tube diameter and folding of the inner layer by drawing out the floats.

**Fig. 4:**
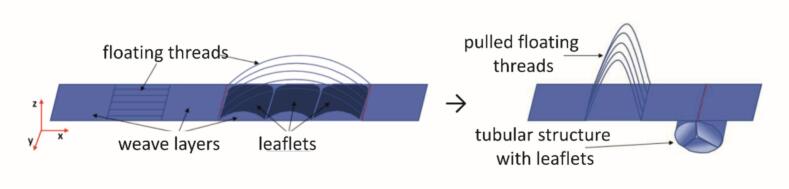
Model tube fabrication based on flat woven fabrics. a) Exposed floating warp threads, b) drawn-in floating warp threads.

A similar method is used to form the internal leaflets. As shown in Fig. 4, two fabric layers are woven in parallel. After forming a pocket, both layers are joined to the outer shell, creating a fixed connection. Floatings across the leaflets enable later erection: pulling these yarns draws the edges of the intermediate layer together (red marked), forming a pleat. This pleat is then anchored to the outer tube using additional floatings or anchoring yarns arranged at 120°, forming a three-pocket valve. The lengths of the seams, fixed at 120°, are approximately equal twice the radius, mirroring the geometry of the native aortic valve.

For the mitral valve (bicuspid, with two leaflets), the geometry was designed to ensure that leaflet seam lengths correspond precisely to the respective outer tube sections. The woven pouches form the final leaflet structures. Once closed, the seams curve naturally and span the valve cross-section closely. To accurately replicate the linear leaflet seams characteristic of a bicuspid valve, additional floating regions were incorporated within the leaflets.

**Fig. 5:**
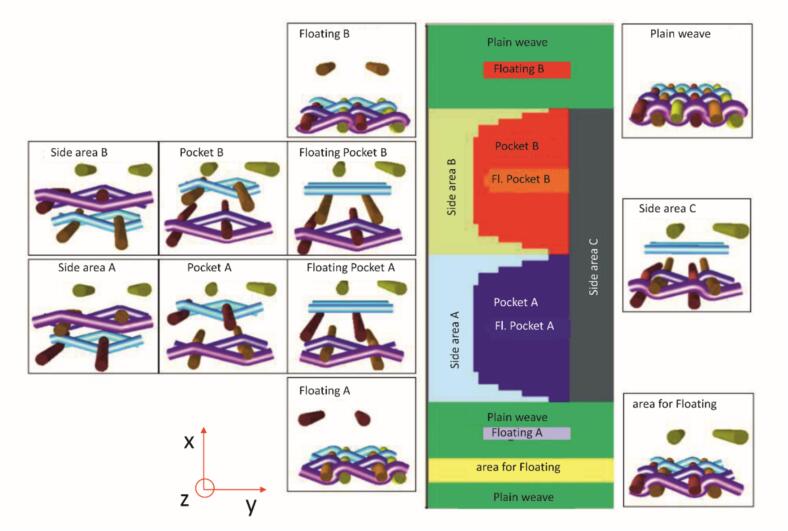
Binding pattern assignment for the different areas according to Concept 1 with two leaflets and regulated seams.

The developed binding concepts enable the flexible fabrication of tubular woven fabrics with integrated valve functionality, allowing systematic variation in leaflet type, number and configuration to closely reproduce both mitral and aortic valve geometries. A patient-specific 3D CAD, unfolded into a 2D plane, was used to generate a colour map that assigns distinct binding patterns to defined regions, as detailed in Chapter 2.4.1. Fig. 5 illustrates this approach for a bicuspid valve, showing linear leaflet seams alongside the corresponding weave patterns for each binding zone.

**Fig. 6:**
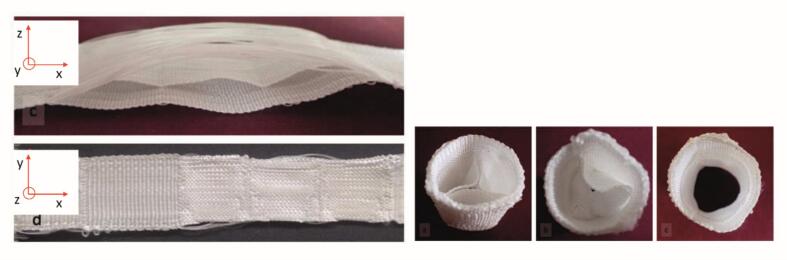
Three-leaflets structure with floating threads (left) and formed structure (right) in closed (a, b) and open states (c)

The weave structures developed along the process chain were produced on a shuttle loom and subsequently shaped by drawing out the float threads. Fig. 6 shows an exemplary woven and formed structure featuring two leaflets.

### 3.2 Mechanical evaluation of the woven heart valve

Mechanical testing demonstrated that the textile heart valve fulfils essential criteria for structural integrity, elastic recovery and functional performance under physiological conditions.

To assess pressure response and valve dynamics, the vascular graft test setup at ITM was upgraded. Through pairwise comparison and utility analysis (VDI 2225), a peristaltic pump (DOSE IT, Integra Biosciences AG) was selected as the most suitable system for generating reproducible pulsatile flow. The setup features a transparent, 3D-printed chamber for precise valve integration and accommodates various pumps and blood-analog test fluids. A custom holder ensures stable implant positioning within defined flow channels.

**Fig. 7:**
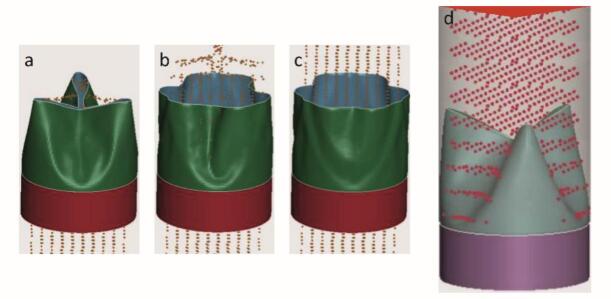
SPH simulation of textile valve: a–c) valve opening; d) fluid retention during backflow.

To address the permeability observed in initial PET samples, valves were from textured multifilament yarns (100 dtex, 0.78 dtex/filament, 57 filaments/cm), improving hydrodynamic performance and reducing actuation pressure. Smoothed Particle Hydrodynamics (SPH) simulations confirmed accurate opening dynamics, controlled flutter and effective backflow prevention (Fig. 7).

The mechanical response closely corresponded to the designed binding architecture: dense regions provided anchoring and structural stability, whereas lighter weaves in leaflet zones enabled flexible motion. These findings demonstrate that material selection, binding configuration and precise process control allow targeted tuning of implant performance.

### 3.3 Reproducible design and manufacturing workflow for textile-based implants

Converting complex anatomical shapes into functional textiles required an integrated, digital driven workflow. CT datasets were segmented in Amira and subsequently imported into SolidWorks, where the implant geometry was divided into functional zones, such as annulus, leaflets and commissures. For each zone, specific mechanical targets, including bending stiffness, compliance, were translated into textile parameters.

**Fig. 8:**
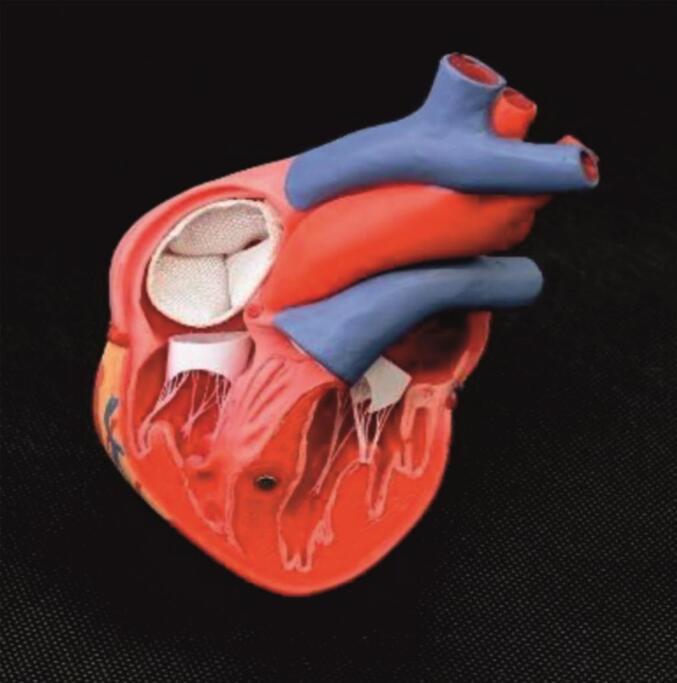
*Fiber-based demonstrator of a seamless, multi-layer tri-leaflet heart valve with integrated functionality* Microscopic analysis confirmed fidelity to the digital design, including multilayer leaflet structures and accurate fiber orientation at commissures. Mechanical tests, comprising uniaxial tension bending measurements, verified that the mechanical targets for each functional zone were achieved. Overall, this workflow enabled the production of anatomically precise and reproducible textile implants with zone-specific mechanical properties.

Binding architectures were developed in EAT DesignScope by selecting and adapting suitable base weaves. Multiple weft layers were overlaid and selectively interlocked to form reinforcement zones and internal cavities (e.g., tri-leaflet dome) within a single continuous weave. The narrow-width MAGEBA loom was modified with four independently tensioned warp beams, an electronic Jacquard for precise yarn control and a custom 3D forming insert that shaped the leaflets during weaving. In addition, a variable-reed beater allowed the creation of physiologically curved geometry without the need for post-forming. Fig. 8 illustrates the fiber-based demonstrator of the seamless, multilayer tri-leaflet heart valve with integrated functionality.

## 4 Discussion

This study demonstrates that woven textile technologies offer a firm basis for next-generation biomedical implants, delivering anatomically precise, mechanically tuned and functionally integrated heart valve constructs tailored to cardiac biomechanics.

Contemporary cardiovascular implants include biological valves (e.g. bovine or porcine pericardium) and mechanical prostheses composed of polymers or metal alloys. Biological valves remain prone to degeneration and calcification, especially in younger patients, often necessitating repeat interventions, as confirmed by recent findings on accelerated valve degeneration in youth [43].

Mechanical valves, while durable, impose lifelong anticoagulation and cannot accommodate growth. In contrast, the woven valve combines mechanical strength, elastic tunability and patient-specific geometry. Moreover, the integration of leaflets and functional zones during weaving eliminates the need for composite assembly or adhesives, simplifying fabrication, reducing costs and enhancing durability, an advantage particularly pertinent in pediatric or anatomically complex cases.

A key innovation lies in the one-step integration of multiple functions. Embedded shape-memory alloys and polymeric elements allow programmed deformation and self-deployment, while zone-specific coatings offer tailored surface functionality. This contrasts with conventional implants that rely on post-processing or modular assembly. This approach enables simultaneous design and fabrication of mechanical and biological domains, streamlining production and improving reproducibility. This aligns with recent advantages in additive manufacturing and biofabrication that emphasize multi-functionality and integrated design from the start [44,45].

Textile engineering inherently supports hierarchical design, from fibers level to woven structures, enabling finely tuned gradients in mechanics and porosity. The CAD-to-textile workflow supports scalable, personalized implants, particularly valuable in reoperation situations, congenital defects, or hybrid procedures. Utilizing clinically familiar materials may also ease regulatory approval, a critical consideration given evolving biocompatibility standards and the high costs associated with regulatory compliance [46].

Nevertheless, significant challenges persist. Long-term biocompatibility, blood-surface interactions and degradation under dynamic physiological conditions require rigorous assessment. Recent data underscore that implant longevity is fundamentally limited by immune-driven calcification [43] and residual antigenicity, which may drive early valve failure. Strategies including decellularization and polymeric alternatives show promise, yet require further validation. Standardization of sterilization protocols, packaging and surgical workflows remains essential for clinical adoption, especially for textile-based implants facing a stringent regulatory landscape. Further research should focus on comprehensive preclinical studies and targeted optimization of surface functionality to minimize thrombogenicity and ensure the long-term performance of these implants.

Technological barriers also remain. Scaling complex binding patterns to diverse anatomical geometries and automating zone-specific designs present manufacturing and engineering challenges. Enhancing the digital structure-function matrix and applying machine learning for automated design optimization should streamline development. Notably, moving beyond static implants, recent literature on 4D fabrication demonstrates the potential of shape-changing constructs responsive to physiological stimuli, a direction highly relevant to future textile-based, self-adapting valve systems [47]. In summary, this study elucidates the translational potential of textile engineering for cardiovascular implants. By integrating anatomical precision, functional layering and streamlined, reproducible fabrication, this modular approach establishes a solid foundation for the next generation of adaptable, biologically integrated implantable devices, provided that ongoing efforts address long-term biocompatibility, manufacturability and regulatory throughput.

## 5 Conclusion

This study demonstrates that woven textile technologies provide a versatile and high-performance foundation for the next-generation of cardiovascular implants. The developed heart valve integrates anatomical precision, mechanical tuning, and functional layering in a single manufacturing step, eliminating the need for composite assembly and enabling patient-specific adaptation. By directly implementing Leaflet geometry, binding zones and structural gradients during weaving process and by incorporating embedded shape-memory alloys and polymer actuators, active deformation and self-deployment are achieved. Zone-specific collagen coatings promote targeted biological integration, while the modular and hierarchical design space inherent to textile engineering enables precise control from the fiber level to the full woven structure. The CAD-to-textile workflow offers scalable, personalized production capabilities, particularly beneficial for pediatric patients and anatomically complex cases. Compared to conventional biological and mechanical heart valves, this approach addresses key limitations such as restricted durability and the requirement for lifelong anticoagulation. Nonetheless, challenges remain regarding long-term biocompatibility, hemocompatibility, regulatory approval, and manufacturing complexity. Incorporating recent advances in additive manufacturing, biofabrication, 4D fabrication, and AI-driven design optimization could pave the way toward fully integrated, adaptive, and reproducibly manufactured implants. Overall, this work underscores the significant translational potential of textile-based heart valve systems and establishes a robust foundation for their evolution into clinically deployable, durable, and patient-specific solutions, which implications for broader adoption in cardiovascular care and personalized medicine.

## CRediT authorship contribution statement

**Cornelia Sennewald:** Writing – original draft, Visualization, Project administration, Methodology, Data curation, Conceptualization. **Jasmin Pilgrim:** Writing – original draft. **Dilbar Aibibu:** Visualization, Validation, Formal analysis. **Thomas Gereke:** Visualization, Validation, Formal analysis. **Philipp Schegner:** Investigation, Data curation. **Chokri Cherif:** Writing – review & editing, Supervision, Project administration, Funding acquisition.

## Declaration of Generative AI and AI-assisted technologies in the writing process

During the preparation of this work, the author(s) used ChatGPT in order to improve the readability and language of the manuscript. After using this tool/service, the author(s) reviewed and edited the content as needed and take(s) full responsibility for the content of the published article.

## Funding

This work was supported by the Research Association Forschungskuratorium Textil e.V. [IGF-Project 19922] and funded through the project management agency AiF as part of the program for “Industrial Collective Research (IGF)” of the Federal Ministry for Economic Affairs and Energy (BMWi) based on a resolution of the German Bundestag. We grearfully acknowledge the mentioned institutions for providing the financial support.

## Declaration of competing interest

The authors declare the following financial interests/personal relationships which may be considered as potential competing interests:

Chokri Cherif reports financial support was provided by Federal Ministry for Economic Affairs and Energy. This work was supported by several projects. We would like to thank the University Hospitals of Dresden and Würzburg for their collaboration and support. If there are other authors, they declare that they have no known competing financial interests or personal relationships that could have appeared to influence the work reported in this paper.
